# The Relationship Between Time Until Full Weight Bearing After Hip Fractures and Vitamin D Levels in Patients Aged 50 Years and Above

**DOI:** 10.7759/cureus.32918

**Published:** 2022-12-25

**Authors:** Abdullah Altuwairqi, Rahaf Sameer Tammar, Ragad Sameer Tammar, Lama Essa Zidan, Anwaar Nafe Alsatty, Shahad Fahad Bahanan, Abaad F Almutairi

**Affiliations:** 1 Orthopedics and Traumatology, King Abdulaziz University Hospital, Jeddah, SAU; 2 Medicine, King Abdulaziz University, Jeddah, SAU

**Keywords:** king abdulaziz university hospital (kauh), healing process, hip fractures, vitamin d levels (vit d level), recovery after hip fracture

## Abstract

Introduction

Hip fractures are common in the elderly, especially with vitamin D deficiency. Currently, there is a paucity of case-control studies regarding the relationship between the time until full weight bearing of hip fractures and vitamin D levels in Saudi Arabia. Our aim is to determine time until full weight bearing of hip fractures in patients with vitamin D deficiency compared with normal vitamin D in the age of 50 and above.

Materials and methods

This was a hospital-based non-interventional retrospective case-control study conducted among patients with hip fractures aged 50 years and above between January 2017 and April 2021. It was done at King Abdulaziz university hospital, Jeddah, Saudi Arabia. A review of the medical records and operation records for the relative operation was done to check patients' lab values around the time of the operation and following the documented healing process. Data were analyzed using SPSS version 26 (IBM Inc., Armonk, New York). Correlation analysis was performed using Spearman's test, and a p-value of 0.05 was considered statistically significant.

Results

In our research, 36 patients were participants in the study, with about two-thirds (22) of the participants being female (61.1%). 52.8% of the participants had an age ranging from 71-80 years, with a mean age of 75.66 ± 9.53 years. A non-significant relationship between the time until full weight bearing of hip fractures and the vitamin d levels is demonstrated. By that, the research question was disproved by the given data.

Conclusion

The time until full weight bearing of hip fracture is not significantly related to vitamin D level. In this study, the majority of patients (77.1%) were vitamin D deficient, which raises our concern for vitamin D deficiency to be a major health problem in our society. However, this evidence should be further assessed in larger trials. Additional studies on this topic are recommended to be done.

## Introduction

Hip fractures represent an important healthcare predicament. Hip fractures can affect and lessen the quality of life and considerably increase mortality and morbidity if not effectively treated, and they have generated a lot of issues, such as social costs and medical complications. Nowadays, hip fractures contribute to most of the orthopedic surgeons' workload, and they remain a cause of about one-quarter of the elderly fractures that require in-hospital admission [1]. To demonstrate the global burden of hip fractures, according to a few studies done, the incidence of hip fractures is estimated to reach 6.3 million in 2050 [2].

Furthermore, based on an assessment done by the Saudi Arabia Ministry of Health, it was estimated that the percentage of occupied beds with hip fracture patients represents 12.82%. The population included four million Saudi nationals (with about 2,031,601 men and 1,952,734 women) aged 45 years or older [3], demonstrating further burden globally wise.

A recent study done in Saudi Arabia suggests that the incidence of hip fractures, with an approximated number of hip fractures in people over the age of 50 years, was around 2949 in 2015 and is expected to increase nearly seven-fold to 20,328 by 2050 [4]. A population of one billion worldwide have vitamin D deficiency [5], and it is certainly a prominent risk factor for increased mortality and hip fracture-related complications [6]. In 2016, Falcker et al. suggested that about 87% of the patients with hip fractures had vitamin D deficiency, further implying that in patients with vitamin D deficiency, the mortality would be increased [7]. Regarding its impact on hip fractures, evidence proves vitamin D deficiency contributes to bone mass loss [8]. And causes an increased risk of fractures due to defects in bone mineralization [9-10]. Nonetheless, there is insufficient data on how vitamin D levels influence mobility and recovery after a hip fracture surgery in elderly patients in the long term. According to the previously reported data, the purpose of this study is to establish the association between vitamin D levels and recovery from hip surgery.

One of the research gaps seen in other studies is the outdated data. Most of the studies were followed up in 2012 and earlier. Few were uploaded in 2021 and are related to the time of weight bearing of hip fractures. Currently, the majority are focused on the relationship between osteoporosis and hip fractures. Hardly any current studies focus on the clinical aspect of the healing of hip fractures and how it is affected by low vitamin D levels regardless of whether it's in the osteoporotic levels or not. And to include, there's a certain aspect of a population gap related to ethnicities, which is demonstrated by the absence of any studies related to the topic being done in Saudi Arabia.

The purpose of our investigation is to further explore the relationship between the time until full weight bearing and length of hospital stay in relation to hip fractures and vitamin D deficiency in patients aged 50 and above in Jeddah. Our study can provide opportunities for further research and studies related to this topic established across Saudi Arabia, more nation-wise rather than a certain region. While there is not enough research focusing on hip fractures that are associated with vitamin D deficiency in the Saudi community, the aim of this study was to determine the outcome following hip fractures correlating with deficiency of vitamin D among the age of 50 or above.

## Materials and methods

Design

A hospital-based non-interventional retrospective case-control study was done at King Abdulaziz University Hospital, Jeddah, Saudi Arabia, to identify hip fracture patients aged 50 or older and the outcome of hip fractures in vitamin D deficient patients and non-vitamin D deficient patients between January 2017 and April 2021.

Participants 

In this study, we included patients who had a hip fracture (fracture of intra-capsular section of femur, upper epiphysis of femur sup capital, mid-cervical, and base of the neck), who were male patients aged 50 years or above or post-menopausal female patients aged 50 years or above. We excluded patients who were younger than 50 years, patients who had other types of femoral bone fracture, patients who were on drugs that can induce osteoporosis, and female patients who were premenopausal or on hormonal replacement therapy. We also excluded patients who were deceased at the time of the study, and patients who did not respond when called repeatedly.

Sample size and sampling technique

In Saudi Arabia, there are approximately 8,768 femoral fractures every year. A sample size with a confidence interval of 95% and a margin of error of 4% is 562 (calculated by an equation), building on the same method and giving that the yearly femoral fractures cases in King Abdulaziz University Hospital. However, the cases of hip fractures in KAUH do not exceed 500 cases, so the sample size for this study is 33-40 cases [11]. Patients were divided into two groups: hip fracture patients that were vitamin D deficient, and hip fracture patients that had normal vitamin D levels.

Data collection methods

Medical records were reviewed to include the desired sample for the study, all patients were contacted to obtain oral consent, and those who couldn't be reached were excluded from the study. Medical and operation records were reviewed again to check patients' lab values around the time of the operation. Not all patients had a medical record that documented vitamin D levels; those patients were contacted to enquire about their vitamin D status. After collecting the needed information, the documented healing process was followed for each of them, and X-ray records and time of weight bearing were reviewed.

Ethical considerations

Consent was obtained from all participants orally, and data remained anonymous. Patients who couldn't be reached were excluded from the study. All the participants had the right to withdraw at any timeline of the study. 

Statistical analysis

Data analysis was done by using SPSS version 26 (IBM In., Armonk, New York). Qualitative data were presented by numbers and percentages, while the Chi-squared test (χ2) was used to evaluate the variables and the relationship between them. Quantitative data was expressed as mean and standard deviation (mean ± SD), and one-way ANOVA, Kruskal Wallis tests, and Mann-Whitney tests were used according to data normality. By using Spearman's test, correlation analysis was performed, and the statistical significance was considered to be a p-value of 0.05.

## Results

A total of 36 participants were involved in our study sample, females were the most prevalent with a percentage of 61.1%, and males were 38.9% of patients (Table 1).

**Table 1:** Frequency for both genders

Gender	Frequency	Percent
Female	22	61.1
Male	14	38.9
Total	36	100

There were two sites of fractures; the most common site of fracture was the neck of the femur with a percentage of 55.6% while the percentage of the intertrochanteric site of fracture was 44.4% (Table 2). Also, the most common type of surgery in our sample was hemiarthroplasty, with 33.3%.

**Table 2:** Frequency of the site of fracture

Site of the fracture	Frequency	Percent
Intertrochanteric	16	44.4
Neck of the femur	20	55.6
Total	36	100

Moreover, our results demonstrate that the patients with low vitamin D levels are more than the patients with normal vitamin D levels. As seen in Table 3, low vitamin D group account for 75% and the normal vitamin D level group account for of 25%.

**Table 3:** Distribution of data according to vitamin D status

Vitamin D levels	Frequency	Percent
Normal	9	25
Low	27	75
Total	36	100

In our study, the minimum length of stay at the hospital was five days, and the maximum was 72 days with an average of 14.72 days, and the time until full weight bearing after the surgery ranged from less than a week up to more than one year, the minimum was four days, and the maximum was 547 days with the mean of 95.36 days, as seen in Table 4.

**Table 4:** Descriptive statistics of the age at time of fracture, length of hospital stays, and time until full weight bearing after the surgery

Descriptive statistics	N	Minimum	Maximum	Mean	SD
Age at the time of fracture	36	50	95	75.58	9.409
Length of stay in hospital (days)	36	5	72	14.72	11.754
Time of full weight bearing after the surgery (days)	36	4	547	95.36	124.787

The results of our analysis can be seen in Table 5, demonstrating a non-significant relationship between vitamin D level and time until full weight bearing, with a p-value of 0.288. Also, there was a non-significant relationship between age at the time of fracture and vitamin D level; the p-value was 0.370. In our analysis, we utilized an independent t-test to compare the two groups of vitamin D levels.

**Table 5:** Independent sample t-tests

Independent sample t-test		Levene's test for equality of variances					t-test for equality of means		95% confidence interval of the difference	
		F	Sig.	t	df	Sig. (2-tailed)	Mean difference	Std. error difference	Lower	Upper
Age at the time of fracture	Equal variances assumed	0.842	0.365	-0.908	34	0.37	-3.296	3.631	-10.675	4.082
	Equal variances not assumed			-0.794	11.322	0.444	-3.296	4.151	-12.402	5.809
Length of stay in hospital (days)	Equal variances assumed	0.332	0.568	-0.275	34	0.785	-1.259	4.585	-10.577	8.059
	Equal variances not assumed			-0.343	22.081	0.735	-1.259	3.674	-8.877	6.358
Time of full weight bearing after the surgery (days)	Equal variances assumed	5.965	0.02	-1.08	34	0.288	-51.741	47.917	-149.12	45.638
	Equal variances not assumed			-1.622	33.392	0.114	-51.741	31.903	-116.619	13.138

## Discussion

To begin with, a brief explanation of the hip fracture normal healing process: the healing process is divided into four stages. The first stage is the formation of a hematoma (days one to five). During the fracture, the arteries of the bone and periosteum are torn, resulting in the formation of a hematoma around the fracture site. During days 5-11, the second stage, which is the callus formation, begins. The release of vascular endothelial growth factor (VEGF) causes angiogenesis, and fibrin-rich granulation tissue begins to grow within the hematoma. Formation of bony calluses (days 11-28) is the third stage at which the endochondral ossification of the cartilaginous callus commences. The last stage is bone reconstruction (day 18 onwards, lasting months to years). The hard callus undergoes recurrent remodeling [12-13].

In light of that, our case-control study aimed to find the correlation between the time until full weight bearing after hip fracture and the vitamin D level among people around 50 years old and above. We compared the time until full weight bearing of those who have normal vitamin D levels and vitamin D deficient patients. We used to measure the healing and recovery of the hip fracture by patients' self-reported data and retrospective data of the time until full weight bearing, length of hospital stay, and age of patients. 

The results demonstrated a non-significant relationship between the time until full weight bearing in patients with normal vitamin D levels and deficiency in vitamin D. However, we did see patients aged 80-91 years having longer days getting to their full weight bearing after the surgery compared to other age groups. Given the results above, no correlation was found other than the participants' age.

Multiple different studies with various methods were utilized to measure the time until full weight bearing and restoring functions after hip fracture. One study focused on the effect of low vitamin D levels before surgery on functional recovery six months following surgery. They used Harris Hip Score (HHS), Parker Mobility Score (PMS), and 36-Short Form Health Survey (SF36) to assess functional recovery and to evaluate mobility after hip fracture surgery [6]. The result showed that patients with extremely low vitamin D levels demonstrated lower scores of PMS and SF36 PF.

Moreover, a study evaluated the relationship between the vitamin D levels at the duration of hip fracture and its healing depending on delay union clinically and on X-ray four months after fracture; the result revealed that patients with persistent vitamin D deficiency during fracture and four months of follow up have a higher rate only for clinical delay union, while X-ray showed no changes with vitamin D level [14]. A systematic review [15], with a total of 105 studies, was done in vitro and in vivo about the cellular effect of vitamin D on the healing process of the fracture. Results were inconclusive, with inconsistency seen throughout each study. Regardless, some of these studies have proven the vitamin D effect on the cellular process of bone healing. However, the exact mechanism of this influence has not yet been defined [16]. 

In another literature review [15], the authors concluded that vitamin D certainly has a role in fracture healing; however, the available data are still too inconsistent to illuminate how and in what manner it has been linked. Despite some of these studies' results, the relationship between vitamin D deficiency on the process of fracture healing is still in need of more research.

One clinical trial focused on the outcome of post-surgical treatment of hip fractures; they divided the patients into two groups - group A had normal vitamin D3 and received vitamin D3 as a blouse, and group B had low vitamin D3 levels received vitamin D3 in the form of a blouse, and then weekly. The results showed that the group with low vitamin D with supplementation had better outcomes of fracture union after four and eight weeks [17].

In one study in Russia, the prevalence of low vitamin D levels among patients who have a fractured hip was 89% [18]. While the prevalence of hip fractures in Saudi Arabia remains unavailable due to insufficient research, we can still see through a retrospective observational study done in 2014 involving 10,709 patients in Jeddah that the prevalence of vitamin D deficiency was 83.6% over five years [19]. 

## Conclusions

Our study shared similar co-morbidities and results of other studies about vitamin D and time of weight bearing of hip fracture in patients affected. And as mentioned previously in the discussion, one study in Jeddah showed that vitamin D deficiency was high as 83.6% in hip fracture patients. It's clearly a high number of patients, although there weren't enough studies and inconclusive results about vitamin D deficiency and hip fracture in Saudi Arabia, I believe that our research will open an opportunity for more research to be done in Saudi Arabia in the future. In conclusion, our study result shows that a non-significant relationship was found between vitamin D status with time until full weight bearing after the surgery (days), length of hospital stay, and fracture and surgical data.
